# Bisphosphonate-related osteonecrosis of the jaw in metastatic breast cancer patients: a review of 25 cases

**DOI:** 10.1186/s40902-016-0052-6

**Published:** 2016-02-01

**Authors:** Hong-Joon Kim, Tae-Jun Park, Kang-Min Ahn

**Affiliations:** Department of Oral and Maxillofacial Surgery, College of Medicine, University of Ulsan, Seoul Asan Medical Center, 88 Olympic-ro 43-gil, Songpa-gu, Seoul 138-736 South Korea

**Keywords:** Bisphosphonate, Breast cancer, Bisphosphonate-related osteonecrosis of the jaw (BRONJ), Extraction, Dental implant

## Abstract

**Background:**

Intravenous bisphosphonates have been used in metastatic breast cancer patients to reduce pathologic bone fracture and bone pain. However, necrosis of the jaw has been reported in those who received intravenous bisphosphonates. Bisphosphonate-related osteonecrosis of the jaw (BRONJ) is caused by dental extraction, dental implant surgery, and denture wearing; however, it occurs spontaneously. The purpose of this study was to report BRONJ in metastatic breast cancer patients.

**Methods:**

Consecutive 25 female patients were referred from the Department of Oncology from 2008 to 2014 for jaw bone discomfort. Staging of breast cancer, history of bisphosphonate infusion, etiology of BRONJ, and treatment results were reviewed. Average age of the patients was 55.4 years old (38–74). Twelve maxillae and 16 mandibles were involved. Conservative treatments such as irrigation, antibiotic medication, analgesics, and oral gargle were applied for all patients for the initial treatment. Patients who had sequestrum underwent debridement and primary closure.

**Results:**

The etiologies of BRONJ were dental extraction (19 cases), dental implant (2 cases), and endodontic treatment (1 case). However, three patients did not have any risk factors to cause BRONJ. Three patients died of progression of metastasis during follow-up periods. Surgical debridement was performed in 21 patients with success in 18 patients. Three patients showed recurred bone exposure and infection after operation.

**Conclusions:**

Prevention of the BRONJ is critical in metastatic breast cancer patients. Conservative treatment to reduce pain, discomfort, and infection is recommended for the initial therapy. However, if there is a sequestrum, surgical debridement and primary closure is the key to treat the BRONJ.

## Background

Currently, breast cancer is an increasing cause of cancer-induced deaths in the world [1]. Multimodality treatment strategies have been proposed for eradicating breast cancer, but still, many patients with breast cancer are in multiple threats to their lives [2]. The bone is the most vulnerable site for breast cancer metastasis. Bone microenvironment has a significant role in harboring disseminated tumor cells and a source of late relapse [3]. Therefore, agents that affect bone metabolism might provide meaningful reductions in the risk of metastasis as well as prevent the development of bone lesions [4]. Malignancy and cancer treatment-induced bone loss can make bone mineral reserves decrease, and patients would face risks of skeletal-related events (SREs) such as pathologic fracture and spinal cord compression [3].

Breast cancer cells usually metastasize to the bone by secreting factors that enable tumor cells to be gathered inside of the bone tissues. Tumor cells produce cytokines that induce osteoclast formation and bone resorption such as interleukin-8 and parathyroid hormone-related protein [2]. Osteoclast would increase both osteolysis and the release of tumor-promoting growth factors from the bone matrix. Using antiresorptive agents to cease osteolysis should make the bone microenvironment difficult for cancer stem cell survival and growth [2].

In patients with bone metastases or multiple myeloma, bisphosphonates are part of the standard treatments [2, 5]. Bisphosphonates are analogs of inorganic pyrophosphates, commonly used for the management of metastatic bone disease such as breast cancer, prostate cancer, and multiple myeloma. Bisphosphonates are capable of localizing metastatic lesions and inhibiting osteoclastic function [6–8]. Because bisphosphonates bind strongly to exposed bone mineral around resorbing osteoclasts, high levels of bisphosphonate in the resorption lacunae would remain even after several years. Within the bone, bisphosphonates are not metabolized and these high concentrations will be held for long periods of time [6]. Although whole mechanism of this bisphosphonate-related osteoclast inhibition has not been completely explained, it has been considered that these compounds affect bone turnover at various levels [9]. On a cellular level, the bisphosphonates target the osteoclasts and may inhibit their function as follows: hindering the osteoclast recruitment; reducing the osteoclast life span [10, 11]. At a molecular level, it has been supposed that bisphosphonates regulate osteoclastic function by interacting with a cell surface receptor or an intracellular enzyme [12]. About 300,000–500,000 cancer patients were prescribed intravenous (IV) bisphosphonates in 2004 [13].

The metastatic breast cancer patients who need for bisphosphonate therapy should be relieved from the bone pain and hypercalcemia and improve quality of life [6]. Bisphosphonate therapy has shown dramatic effect on reducing the risk of SREs by reducing this risk by nearly one third, and because of this reason, intravenous bisphosphonates are commonly used in the oncology practice [14, 15]. Zolendronate, the most potent bisphosphonate, is the second-generation bisphosphonate and approved for patients with metastatic breast cancer, multiple myeloma, hypercalcemia of malignancy, or Paget’s disease of bone and for patients with documented bone metastases from any solid tumor (i.e., prostate cancer, lung cancer) [6]. In comparison with other bisphosphonates, zolendronate is crucially more effective in reducing the risks of SREs and controlling hypercalcemia of malignancy [16].

Bisphosphonate-related osteonecrosis of the jaw (BRONJ), first reported by Marx in 2003, is a rare but emerging complication associated with long-term use of bisphosphonates, especially pamidronate and zoledronate [17]. The common symptoms of BRONJ are teeth mobility, swelling, bone dehiscence, chronic bone necrosis, and osteolytic radiographic features. The American Association of Oral and Maxillofacial Surgeons (AAOMS) proposed a staging system to suggest guidelines for prognosis and treatment of BRONJ (Table 1) [18]. There are several reports regarding breast cancer with jaw bone necrosis [3, 19–21]; however, no case review has been reported in our country. In the present study, we tried to evaluate the BRONJ in breast cancer patients who were treated with intravenous BPs that whether conservative or surgical management worked on each stage.

**Table 1:** Staging of bisphosphonate-related osteonecrosis of the jaw

Stages	Description
At risk	No apparent necrotic bone in patients who have been treated with either oral or intravenous bisphosphonates.
Stage 0	There is no clinical evidence of necrotic bone, but there are nonspecific clinical findings and symptoms such as swelling of the soft tissue and fistula formation.
Stage 1	There is exposed and necrotic bone or fistulas that probes to the bone in asymptomatic patients but there is no evidence of infection.
Stage 2	There is exposed and necrotic bone or fistulas that probes to the bone associated with infection as evidenced by pain and erythema in the region of exposed bone with or without purulent drainage.
Stage 3	There is exposed and necrotic bone or a fistula that probes to the bone with pain, infection, and one or more of the following: exposed and necrotic bone extending beyond the region of alveolar bone resulting in (1) pathologic fracture, (2) extraoral fistula, (3) oral-antral/oral-nasal communication, or (4) osteolysis extending to the inferior border of the mandible or sinus floor.

## Methods

A total of 25 female BRONJ patients who suffered from breast cancer with bone metastasis from January 2008 to November 2014 were included in this study. All patients had received zolendronate (Zometa**®**, Novatis, USA) for treating metastatic bone lesions. Average age was 55.4 years old (38–74). Twelve maxillae and 16 mandibles were involved. The diagnosis of BRONJ in these patients was based on the guidelines provided by the AAOMS position paper (Table 1) [18]. Staging of the breast cancer, duration of bisphosphonate usage, etiology of BRONJ, and treatment results were reviewed retrospectively. Conservative treatment with irrigation, antibiotic medication, analgesics, and oral gargle was applied for all patients for the initial treatment. Patients who had a sequestrum underwent debridement and primary closure. Because of the retrospective study with de-identification of the patient’s data, institutional review board of our institution exempted ethical review of this study.

## Results

### Staging and treatment of breast cancer

Staging of the breast cancer at initial visit, past treatment history about surgery, chemotherapy and radiation, and survival of breast cancer patients are summarized (Table 2). The staging of breast cancer patients was based on the guidelines provided by American Joint Committee on Cancer (AJCC) staging manual (7th edition) [22]. Survival period means the months from operation date or the day that biopsy proved malignant breast cancer when operation did not perform to the latest follow-up. According to AJCC cancer staging manual, two (8 %) were stage 1, eleven (44 %) were stage 2, three (12 %) were stage 3, and nine patients (36 %) were stage 4. Nine patients underwent chemotherapy without surgery. The mean follow-up period was 16.0 months (2–65 months) and mean survival period of patients was 96.2 (10–240). Three patients died of progression of metastasis during follow-up periods.

**Table 2:** Staging, treatment, and survival of metastatic breast cancer patients

Case number	Age	Sex	Stage	Operation (Y/N)	Chemotherapy (Y/N)	Survival (Y/N)	Survival period (months)
1	45	F	IIA	Y	Y	Y	118
2	53	F	IV	N	Y	Y	36
3	58	F	IC	Y	N	Y	105
4	55	F	IIB	Y	Y	Y	76
5	70	F	IIA	Y	Y	Y	240
6	59	F	IIB	Y	Y	Y	39
7	67	F	IV	N	Y	Y	104
8	50	F	IIA	Y	N	Y	139
9	61	F	IV	N	Y	N	42
10	51	F	IIIA	Y	Y	Y	149
11	55	F	IV	N	Y	Y	44
12	52	F	IV	N	Y	Y	64
13	67	F	IIIA	Y	Y	Y	93
14	38	F	IIIC	Y	Y	N	132
15	65	F	IIB	Y	Y	Y	175
16	49	F	IV	N	Y	N	19
17	49	F	IIB	Y	Y	Y	124
18	48	F	IIA	Y	Y	Y	135
19	70	F	IIA	Y	Y	Y	150
20	42	F	IV	N	Y	Y	10
21	49	F	IIA	Y	Y	Y	94
22	48	F	IIA	Y	Y	Y	124
23	55	F	IV	N	Y	Y	23
24	54	F	IV	N	Y	Y	86
25	74	F	IA	Y	Y	Y	85

*F* female, survival period means the months from operation date or the day that biopsy proved malignant breast cancer when operation did not perform to the last follow-up

### Clinical features and medical history

Initial symptoms were pain in sixteen patients (64 %), swelling in seven (28 %), pus discharge in eight (32 %), tooth mobility in two (8 %), unhealed operation site in three (12 %), intraoral fistula in one (4 %), while multiple symptoms were observed in individuals (Table 3). Mandible was involved in 16 patients and maxilla in 12 patients. Three patients were affected both mandible and maxilla. The etiologies for BRONJ were mainly tooth extraction in nineteen patients (76 %), dental implant in two (8 %), endodontic treatment in one (4 %), and spontaneously occurred in three patients (12 %). Based on the BRONJ classifications of AAOMS position paper, one patient (4 %) was stage 3, sixteen (64 %) were stage 2, one (4 %) was stage 1, and six (24 %) were stage 0. All of the patients had received intravenous bisphosphonate therapy with 4 mg of zolendronate every month. Mean number of Zometa**®** injection was 32.7 (3–114) times. In the aspect of comorbidity, 3 of 25 patients were affected by diabetes mellitus and 4 were affected by hypertension.

**Table 3:** Clinical features and bisphosphonate history of patients

Case number	Age	Sex	Chief complaint	Location	Trigger event	Stage	BP therapy	Dose	Injection times	Comorbid disease
1	45	F	Itching sensation	Maxilla	Extraction	1	Zolendronate	4 mg/monthly	44	-
2	53	F	Pain, swelling	Maxilla	Extraction	2	Zolendronate	4 mg/monthly	41	Hypertension
3	58	F	Pain, Swelling	Mandible	Endo	2	Zolendronate	4 mg/monthly	3	-
4	55	F	Swelling, unhealing extraction socket	Mandible	Extraction	0	Zolendronate	4 mg/monthly	7	-
5	70	F	Pain, swelling, pus discharge	Mandible	Implant	2	Zolendronate	4 mg/monthly	18	Diabetes mellitus, hypertension
6	59	F	Pain, pus discharge	Maxilla	Extraction	2	Zolendronate	4 mg/monthly	20	Hypertension
7	67	F	Pain, pus discharge	Maxilla	Extraction	0	Zolendronate	4 mg/monthly	24	Hypertension
8	50	F	Pus discharge, unhealing implantation site	Mandible	Implant	0	Zolendronate	4 mg/monthly	114	-
9	61	F	Pain, swelling, pathologic fracture	Mandible	Extraction	3	Zolendronate	4 mg/monthly	7	Diabetes mellitus
10	51	F	Pain	Maxilla	Extraction	2	Zolendronate	4 mg/monthly	49	Diabetes mellitus
11	55	F	Pain	Mandible	Extraction	2	Zolendronate	4 mg/monthly	14	-
12	52	F	Tooth mobility	Mandible	Extraction	0	Zolendronate	4 mg/monthly	67	-
13	67	F	Pain, tooth mobility	Bilateral mandible	Extraction	2	Zolendronate	4 mg/monthly	20	-
14	38	F	Pus discharge	Maxilla	Extraction	2	Zolendronate	4 mg/monthly	21	-
15	65	F	Pus discharge	Maxilla	Extraction	2	Zolendronate	4 mg/monthly	23	-
16	49	F	Pus discharge	Mandible	Extraction	0	Zolendronate	4 mg/monthly	13	Hypothyroidism
17	49	F	Pain	Mandible	Extraction	2	Zolendronate	4 mg/monthly	58	-
18	48	F	Pain, swelling	Maxilla and mandible	Extraction	2	Zolendronate	4 mg/monthly	47	-
19	70	F	Pain, unhealing extraction socket	Bilateral maxilla and mandible	Extraction	2	Zolendronate	4 mg/monthly	52	-
20	42	F	Pus discharge	Maxilla and mandible	Extraction	2	Zolendronate	4 mg/monthly	40	-
21	49	F	Pain	Maxilla	Spontaneous	0	Zolendronate	4 mg/monthly	9	-
22	48	F	Pain, pus discharge	Mandible	Extraction	2	Zolendronate	4 mg/monthly	43	-
23	55	F	Pain	Mandible	Spontaneous	2	Zolendronate	4 mg/monthly	10	-
24	54	F	Pain	Maxilla	Extraction	2	Zolendronate	4 mg/monthly	48	-
25	74	F	Swelling, intraoral fistula	Mandible	Spontaneous	2	Zolendronate	4 mg/monthly	25	-

*F* female

### Treatment and outcome for BRONJ

All 25 patients were treated conservatively with antibiotics, chlorohexidine gargle, and analgesics at the time of initial visit. Surgical treatment was performed in 21 patients (Table 4). Most of the patients required sequestrectomy and saucerization. Two patients underwent simple curettage and one underwent dental implant fixture removal. Four patients (16 %) were managed by conservative treatment solely. When BRONJ was diagnosed, patient had been recommended to stop administration of zolendronate except one who suffered from bone metastasis on mandible (No. 12 patient). Systemic condition and intraoral and extraoral characteristics were assessed in collaboration with medical oncologists. A surgical approach was considered after 3 months of bisphosphonate discontinuation in patients with chronic symptoms. In this study, surgical treatment was performed in 21 patients (84 %) with success in 18 patients. Three patients showed repeated bone exposure and infection after initial operation. Healing of the oral mucosa was observed in 19 patients (76 %) with no other signs.

**Table 4:** Treatment and outcome of patients

Case number	Age	Sex	Surgical treatment	Follow-up (M)	BP discontinuation	Outcome
1	45	F	Sequestrectomy	19	Yes	Healed mucosa
2	53	F	Sequestrectomy	5	Yes	Healed mucosa
3	58	F	Sequestrectomy	12	Yes	Another bony exposure (Mx), death
4	55	F	Conservative management	12	Yes	Healed mucosa
5	70	F	Sequestrectomy	65	Yes	Healed mucosa
6	59	F	Conservative management	2	Yes	No more follow-up with unhealed state
7	67	F	Curettage	12	Yes	Healed mucosa
8	50	F	Implant removal	17	Yes	Bony exposure
9	61	F	Segmental mandibulectomy	36	Yes	Death
10	51	F	Sequestrectomy	18	Yes	Healed mucosa
11	55	F	Sequestrectomy	25	Yes	Healed mucosa
12	52	F	Conservative management	19	No	Mandible metastasis, skin fistula
13	67	F	Sequestrectomy	11	Yes	Healed mucosa
14	38	F	Sequestrectomy	6	Yes	Healed mucosa, death
15	65	F	Sequestrectomy	9	Yes	Healed mucosa
16	49	F	Curettage	2	Yes	No more follow-up with healed state
17	49	F	Sequestrectomy	35	Yes	Healed mucosa and skin
18	48	F	Sequestrectomy	6	Yes	Bony exposure
19	70	F	Sequestrectomy	21	Yes	Healed mucosa
20	42	F	Sequestrectomy	10	Yes	Healed mucosa
21	49	F	Conservative management	10	Yes	Healed mucosa
22	48	F	Sequestrectomy	4	Yes	Healed mucosa
23	55	F	Sequestrectomy	13	Yes	Healed mucosa
24	54	F	Sequestrectomy	17	Yes	Healed mucosa
25	74	F	Sequestrectomy	14	Yes	Healed mucosa

*F* female, *BP* bisphosphonate, *M* month, *Mx* maxilla

### Case review

**Fig. 1:**
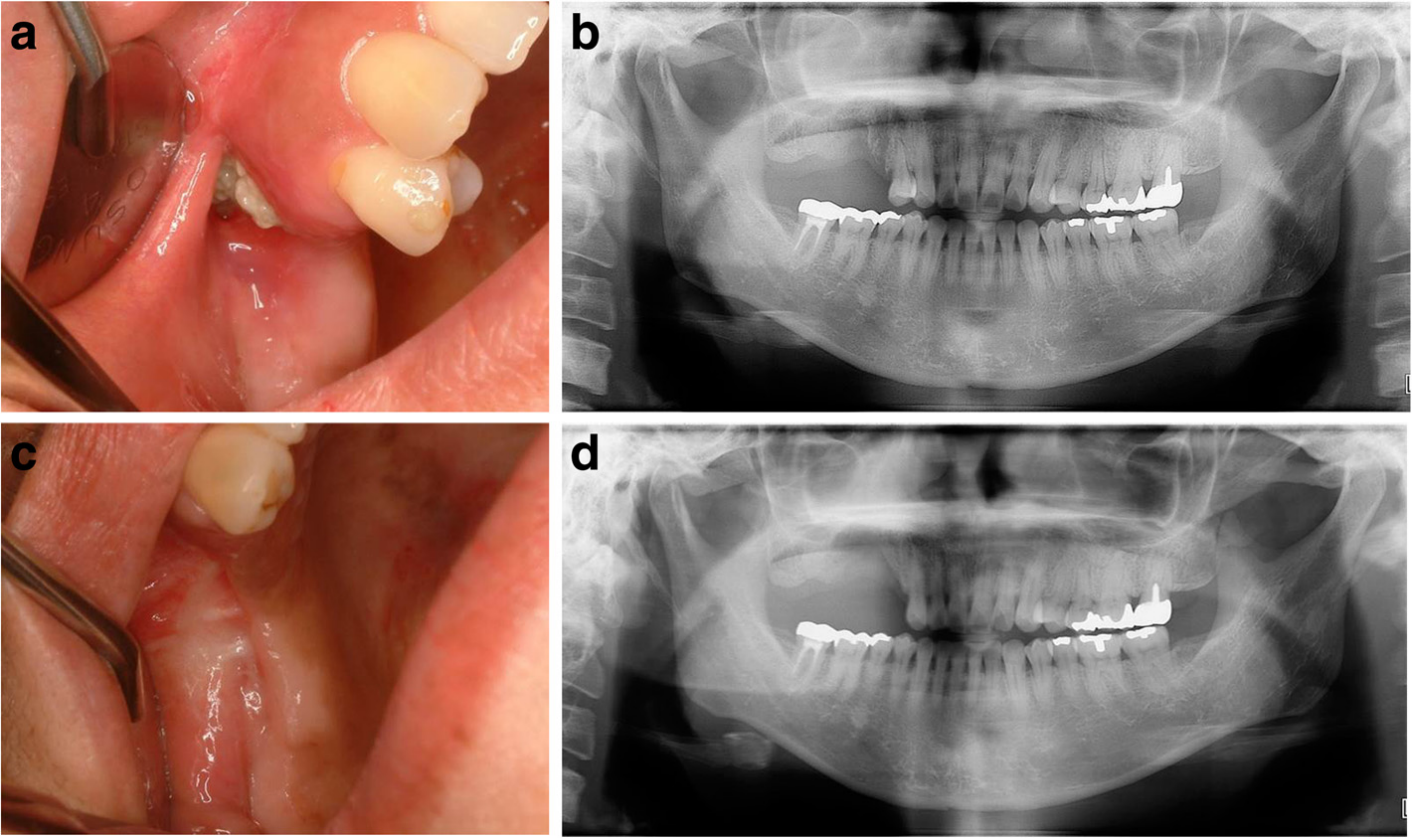
Clinical, panoramic examinations of patient (No. 2 patient). **a** Exposed maxillary bone in the buccal side of second premolar. **b** Initial panoramic view showing bone destruction in the right maxillary premolar area with unhealed extraction socket. **c** Intraoral photograph showing healed mucosa 4 months postoperation. **d** Panoramic view showing bone defect in right maxillary premolar area 4 months postoperation

In September 2014, number 2 patient was referred from the Department of Oncology for maxillary bone pain and gingival swelling after extraction of the right maxillary premolar. Her stage of breast cancer was IV, and she had received chemotherapy for palliative treatment. She had received intravenous bisphosphonate for more than 3 years and had hypertension for comorbidity. Necrotic bone was observed on the buccal side of right upper premolars. After a month of conservative therapy, she underwent sequestrectomy and primary closure with buccal fat graft. Inflamed mucosa and necrotic sequestrum had been treated and all of the clinical symptoms were improved (Fig. 1a–d).

**Fig. 2:**
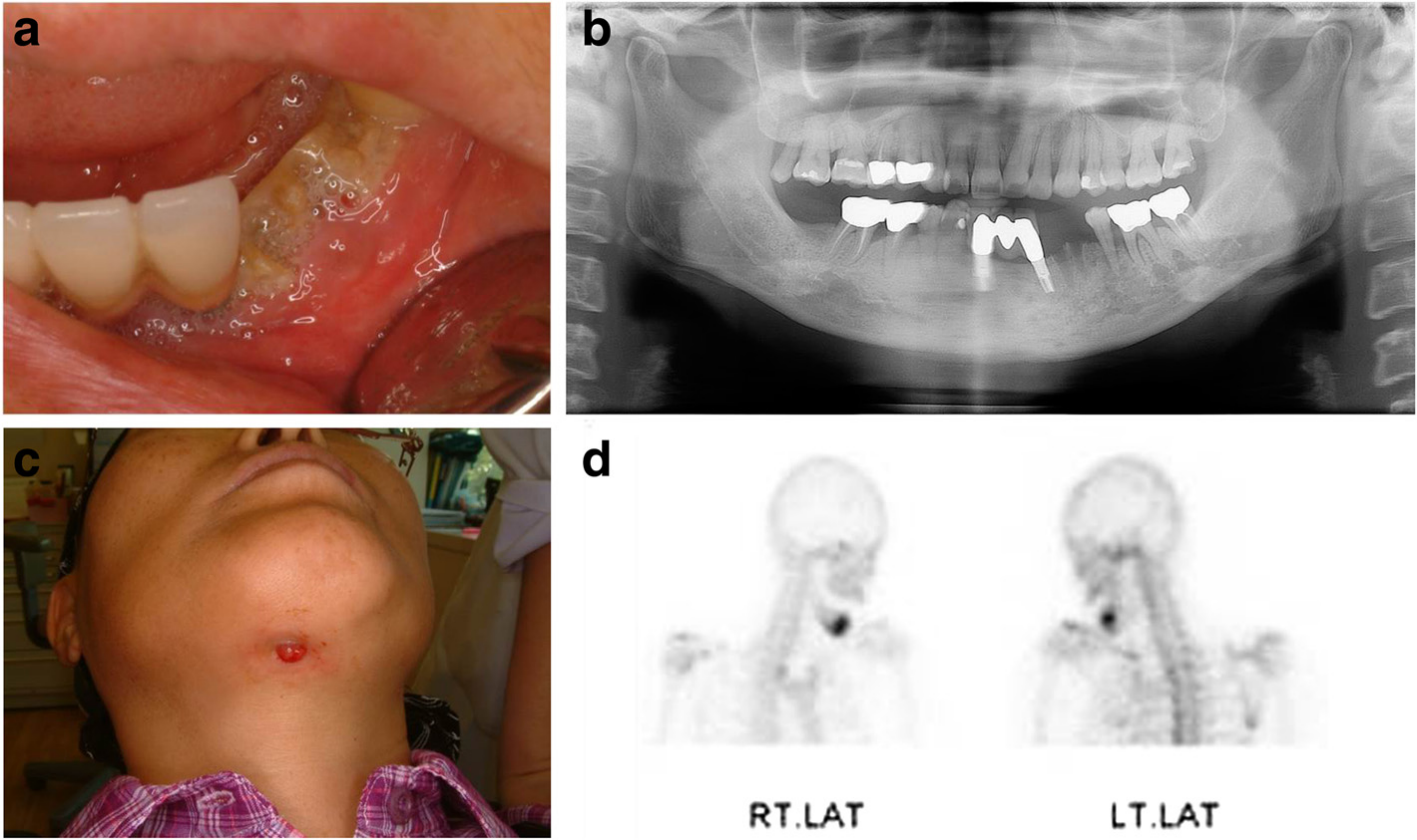
Clinical, panoramic, and bone scan examinations of patient (No. 12 patient). **a** Clinical photograph of exposed mandible. **b** Panoramic image during conservative treatment. **c** Extraoral fistula formation with pus discharge in the right submental area. **d** Bone scan image showing hot uptake in the anterior mandible

Number 12 patient was referred from the Department of Oncology complaining of tooth mobility during chemotherapy. Her stage of breast cancer was also IV, and she did not undergo operation because of multiple bone metastases. She had received chemotherapy for palliative treatment. She had received intravenous bisphosphonates for more than 5 years. According to the bone scan image, hot uptake was found in the anterior mandible which resembled bone metastasis. For differential diagnosis, biopsy was performed before operation resulting in osteomyelitis with bacterial contamination. During conservative treatment, she reported skin fistula and necrotic bone exposure in oral cavity. Due to fast progression of the metastasis, no surgical exploration was performed (Fig. 2a–d).

**Fig. 3:**
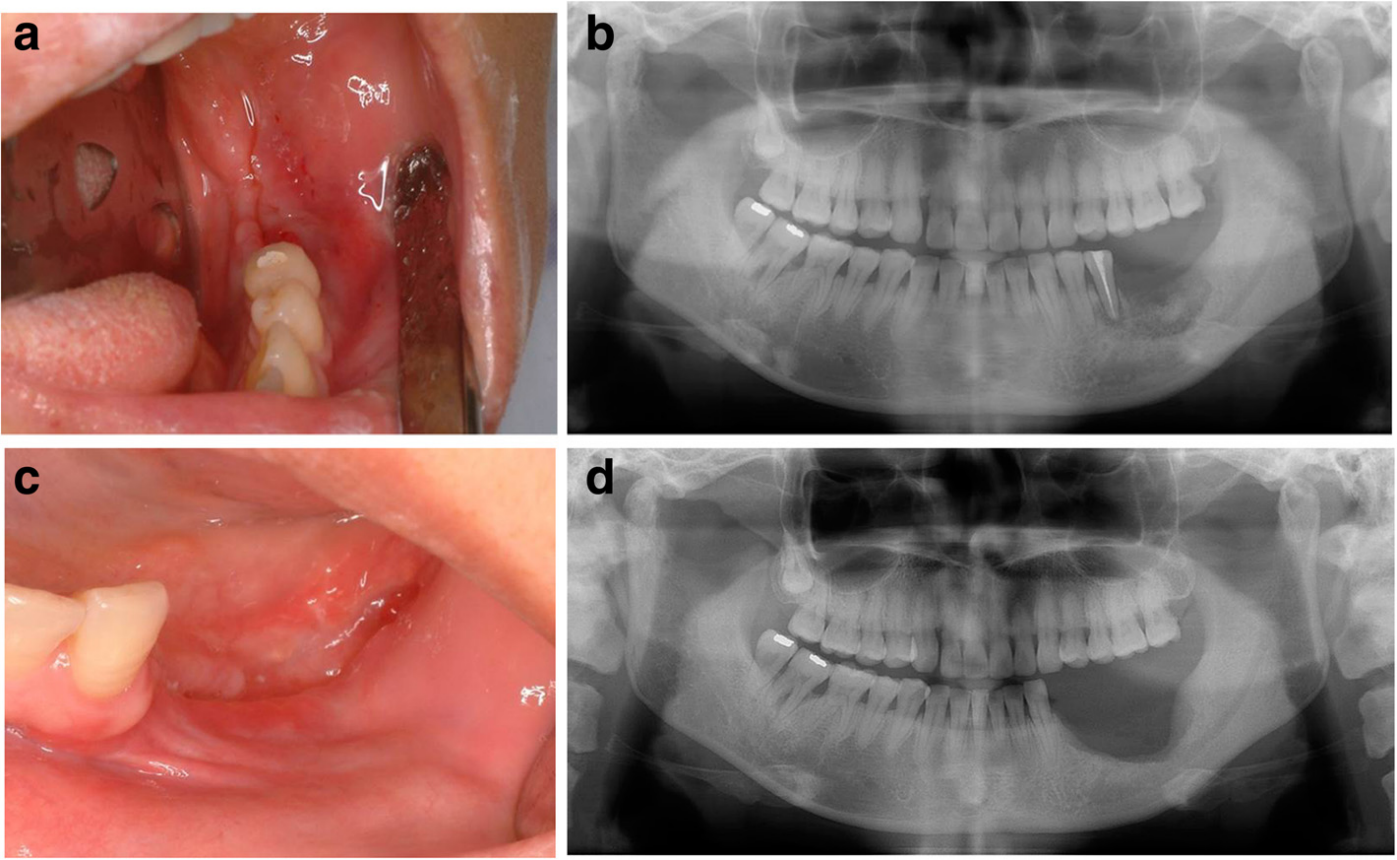
Clinical, panoramic examinations of patient (No. 22 patient). **a** Intraoral photograph showing inflamed mucosa with swelling. **b** Panoramic view showing sequestrum in the left posterior mandible. **c** Intraoral photograph showing healed mucosa 2 months postoperation. **d** Panoramic view showing cortical bone healing and resection of sequestrum 2 months postoperation

Number 22 patient was referred from the Department of Oncology for mandible bone pain and pus discharge on the left lower molar area where extraction had been performed in previous dental clinic. Her stage of breast cancer was IIA, and she underwent operation. She had been treated with intravenous bisphosphonate for more than 3 years after realizing bone metastasis. Sequestrum was observed on panoramic view. For surgical debridement, bisphosphonate was discontinued for 2 months before operation. After surgery, progression of BRONJ ceased without pain and swelling. Two months postoperation, cortical margin around operation site was distinct in panoramic view (Fig. 3a–d).

## Discussion

It has been reported that the incidence of BRONJ with intravenous bisphosphonates is more frequent than that with oral bisphosphonates [23]. The incidence of BRONJ with intravenous bisphosphonates has been reported 0.8–1.2 % on average, increasing up to 21 % after injection of bisphosphonate for 3 years or more [5, 24, 25]. Bisphosphonates bind to bone hydroxyapatite for almost 10 years, and because of this reason, discontinuation of bisphosphonate administration before dental treatment is still disputable [26]. Cancer patients who have high risks for bone pain, hypercalcemia, or pathological fracture could effectively benefit from intravenous bisphosphonates. However, BRONJ could happen during treatment [3, 19–21]. When BRONJ is suspicious, it is highly recommended to stop using them. In this study, 24 patients stopped taking bisphosphonates after consulting with an oncologist. However, it could not be discontinued in one patient who had suffered from multiple bone metastases with hypercalcemia.

The relatively high percentage of the maxilla (12 of 25 patients) involvement of BRONJ in this study is uncommon because the maxilla is provided with rich vascular supply. This finding is distinguished from other studies which reported dominant involvement of the mandible [27–30]. It may be related to the mechanism by which bisphosphonates would not only inhibit the angiogenesis but also affect in other way [18]. One of the most frequent initiations of BRONJ is a dental extraction. In our case series, dental extraction was more frequent in the maxilla than the mandible.

The potent inhibition of osteoclast proceeds to reduce bone resorption and interrupt normal bone turnover remodeling, resulting in reduction of some mechanical properties in skeletal health [31]. As bone resorption occurs, cytokines and growth factors would be released into the surrounding matrixes that are significant for regulating new bone development. The inhibition of new bone formation would lead to degrade bone quality during growth and fracture healing [6]. Systemic conditions of patients involving diabetes mellitus or coagulopathy have also been reported as a risk factor for BRONJ [18, 32]. In the present study, three of the patients had diabetes mellitus.

Bisphosphonate therapy should be delayed until all necessary dental treatments have been performed, except life-threatening hypercalcemia [18]. In this report, dental extraction was the most common etiology for BRONJ initiation. Other surgical procedures such as dental implant surgery and preprosthetic surgical treatment could be the causes of BRONJ [33]. During bisphosphonate therapy, patients should manage their oral hygiene. Invasive dental procedures should be avoided during bisphosphonate therapy, if that is possible. Bacterial infection was observed in all the present cases, therefore, antibiotic treatment should be ensured on BRONJ patients [23].

Recent studies have reported that surgical debridement might have benefited in eradicating necrotic bone in comparison with conservative treatment [27, 34–36]. If invasive dental procedure is determined, a systemic perioperative antibiotic treatment is recommended. Surgical debridement should be done for those patients who complained symptoms. Obtaining a surgical margin with viable bleeding the bone is significant in surgery, and primary closure for wound healing should be served by using mucosal flaps for bone coverage [3]. It is recommended that bisphosphonate administration should be withheld for 6–8 weeks before and after dental procedures [3, 18]. After complete wound healing is achieved, bisphosphonate therapy could be reinitiated because the risk of SREs would still exist and increase during the course of disease [37]. Despite the several efforts for setting guidelines, the optimum treatment for BRONJ remains unclarified. It is necessary to accumulate further clinical data to make the standard for effective treatment in BRONJ patients.

## Conclusions

Prevention of the BRONJ is critical in metastatic breast cancer patients. Dental extraction is the main etiology for BRONJ. Conservative treatment to reduce pain, discomfort, and infection is recommended for the initial therapy. However, if there is a sequestrum which is separated from the basal bone of the jaw, surgical debridement and primary closure is the key to treat the BRONJ.
